# Disgusting odours affect the characteristics of the Adaptive Force in contrast to neutral and pleasant odours

**DOI:** 10.1038/s41598-021-95759-0

**Published:** 2021-08-12

**Authors:** Laura V. Schaefer, Silas Dech, Markus Aehle, Frank N. Bittmann

**Affiliations:** Division Regulative Physiology and Prevention, Department Sports and Health Sciences, University of Potsdam, Karl-Liebknecht-Str. 24-25, 14476 Potsdam, Germany

**Keywords:** Motor control, Olfactory system, Sensorimotor processing, Neurology, Neurophysiology

## Abstract

The olfactomotor system is especially investigated by examining the sniffing in reaction to olfactory stimuli. The motor output of respiratory-independent muscles was seldomly considered regarding possible influences of smells. The Adaptive Force (AF) characterizes the capability of the neuromuscular system to adapt to external forces in a holding manner and was suggested to be more vulnerable to possible interfering stimuli due to the underlying complex control processes. The aim of this pilot study was to measure the effects of olfactory inputs on the AF of the hip and elbow flexors, respectively. The AF of 10 subjects was examined manually by experienced testers while smelling at sniffing sticks with neutral, pleasant or disgusting odours. The reaction force and the limb position were recorded by a handheld device. The results show, inter alia, a significantly lower maximal isometric AF and a significantly higher AF at the onset of oscillations by perceiving disgusting odours compared to pleasant or neutral odours (p < 0.001). The adaptive holding capacity seems to reflect the functionality of the neuromuscular control, which can be impaired by disgusting olfactory inputs. An undisturbed functioning neuromuscular system appears to be characterized by a proper length tension control and by an earlier onset of mutual oscillations during an external force increase. This highlights the strong connection of olfaction and motor control also regarding respiratory-independent muscles.

## Introduction

Olfactomotor control is especially investigated by considering the triggered sniff due to an olfactory stimuli^1^, since the “primary sensory motor component of olfaction is the sniff”^2^. This sniff results from a “rapid and powerful contraction of the diaphragm”^2^. The motor output of respiratory-independent muscles is rarely considered. The present investigation should reduce this lack of knowledge by examining the influence of different odours on the Adaptive Force (AF), which characterizes a special neuromuscular holding function of limb muscles.

The main olfactory system lies in the nasal chambers within a neuroepithelium lining^3,4^. The cranial nerves I (olfactory nerve) and V (trigeminal nerve) mediate the chemosensation of olfaction^4^. Olfactory afferences are firstly transmitted to the olfactory bulb^5^. Subsequently, the olfactory information is processed by primary and secondary olfactory regions^4^. The former includes the piriform cortex, olfactory nucleus and tubercle, amygdala and the entorhinal cortex; the latter involves the hippocampus, hypothalamus, thalamus, orbitofrontal cortex and the cerebellum^4^. Olfactory afferences are projected directly to the piriform cortex and the limbic system (amygdala, hippocampus)^5,6^. Additionally, the cingulate cortex was reported to be associated with olfaction^7^. This highlights the complex connectivity of different central structures involved in olfaction and is an explanatory base of the strong connection of olfaction, emotions and motor control. Ferdenzi et al. suggested that the sniffing behaviour “might be involved in adaptive responses protecting the subject from possible harmful substances”^1^. The olfactory system, therefore, “serves as an early warning system for spoiled food and noxious or dangerous environmental chemicals”^3^. Perceiving emotionally related threatening stimuli, like unpleasant odours, presumably result in direct influences on motor activity^8,9^.

Investigations regarding olfactomotor control revealed a reduction in sniff volume and duration by increasing odour concentration^1,2,10,11^. Furthermore, perceiving a malodour leads to a reduction in sniffing^12^. Not only a real olfactory perception provokes a reaction of motor activity like the sniff, but also an imagery of pleasant odours enhances the olfactomotor activity (sniff) compared to unpleasant odours^13^. A sniffing alone (non-odourant) already activates the anterior cerebellum^14^. Because of the speed of sniff modulation, the olfactomotor sniff feedback control was suggested to be subcortical^2^. According to Johnson et al.^2^ the odourant transduction lasts ~ 150 ms and the “odourant-induced cortical evoked potentials have latencies of around 300 ms”^2^. Also other researchers found olfactory afferences with latencies of around 300 ms^15^.

All those considerations highlight the importance of motor action in the sense of sniffing in odour perception^1^. However, influences of olfaction on the muscles of the extremities in humans were not considered yet to the authors’ knowledge. Derjean et al. suggested a strong coupling between the olfactory system and the reticulospinal cells, which are command neurons for locomotion^16^. In lampreys the activation of this olfactory-motor pathways resulted in swimming movements^16^. In humans, motoric reactions to olfaction are considered, for instance, concerning the startle reflex with respect to pleasant, unpleasant or no odour^17^. Closer to motor control in the above mentioned sense was an investigation showing that lavender odour could reduce falls in elderly nursing home residents^18^. Besides the motor output, other regulating systems are investigated concerning odour induced changes, like the cardiovascular system^19,20^, psychophysiological brain activity^21^, cognition and behaviour^22^ or the influence of odours on the quality of life^23,24^. Hence, there seems to be a lack of knowledge of the effects of different odours on the motor output of respiratory-independent skeletal muscles.

In health, sports and movement sciences or medicine this motor output is mostly measured by pushing against a resistance. Thereby, the participants have to apply a force, but do not have to react to an external force. During a holding isometric action, the reaction to an external force is required. Therefore, it was recently suggested that two forms of isometric muscle action exist: a holding and a pushing isometric muscle action (HIMA; PIMA)^25,26^. Some investigations showed that the duration to maintain a defined force level is significantly briefer during HIMA compared to PIMA^25–30^. Furthermore, differences in the power frequency distribution were present^25,28^. This indicates that HIMA is controlled by more complex control strategies than PIMA. Additionally, it was hypothesized that the control mechanisms of HIMA are closer to the one during eccentric muscle action. In contrast, PIMA was rather interpreted as a stopped concentric muscle action and, therefore, the control strategies might be similar to the ones during concentric muscle action^25,26,31^. Enoka and Duchateau suggested a more complex neuronal control during eccentric muscle actions compared to concentric contractions^32–35^. In case the neuromuscular system has to adapt to a varying external force in the described holding manner or during muscle lengthening, it is reasonable to assume that the requirements regarding the neuromuscular control could be even higher^36,37^. Therefore, it might be beneficial to investigate the Adaptive Force (AF) to challenge the neuromuscular control in a special way^38,39^. The AF reflects the holding capability to adapt adequately to external forces with the intention to maintain a given position or motion^38,40,41^. One specific way to execute the AF is to hold a given position against a rising external load. Thereby, the motor output must be permanently adjusted regarding the sensory input triggered by the external load. This necessitates a sound functionality of the sensorimotor control. If the neuromuscular system is not able to match the increasing external force isometrically, the muscle starts to lengthen in the course of the external force increase^38^. Therefore, the AF can be realized during an isometric or an eccentric muscle action. For the processing of AF, a mixed feed-forward and feedback control is assumed to be necessary. The mixed control was suggested by Caligiore et al.^42^. It is closely related to the “forward model”, which “predicts the behaviour of the motor apparatus for a motor command”^42,43^. This requires an efference copy and direct somatosensory afferences^42,43^. For an optimal execution of AF, the muscle length and joint angle should stay constant for as long as possible during the external force increase. It is hypothesized that the AF reflects the functionality of the complex sensorimotor control and its detection could particularly be suitable to identify interferences in these control circuitries. The cerebellum as well as the ventrolateral thalamus are, inter alia, involved in olfaction^2,14,44^ and both structures are relevant for adaptive motor control. Additionally, olfaction and emotion are evolutionarily strongly coupled^45–47^. The effect of emotions on motor control are secured, e.g., by the role of the hippocampus^48^ and the amygdala^8^. Sagaspe et al. suggested that “threat perception may influence brain system involved in motor control in humans, through partly overlapping but also partly different pathways than those mediating voluntary inhibition”^8^. Therefore, an influence of olfaction on the AF as motor output is conceivable.

The assessment of AF can be performed manually (clinical, with or without a handheld device) or using an equipment system based on pneumatics^39,41^. In each case, the subject should adapt to the external increasing force application with the intention to maintain the position by holding isometrically for as long as possible. The assessment of the AF by the manual muscle test (MMT) can be objectified by recording the kinematics and dynamics using a handheld device. Thereby, the advantages of the flexible and time saving MMT are combined with the requirements of objectivity for scientific purposes. That is why the MMT in the sense of a “break test”^39,49^ was used here to investigate the influence of different odours on the AF.

During the MMT the force maximum (AFmax) can arise during isometric (AFiso_max_) or during eccentric (AFecc_max_) conditions. Thereby, the AFmax is not necessarily equivalent to the MVIC of the participant but refers to the maximum force reached during the MMT either under isometric or eccentric behaviour. Since adjusting the muscular tension by maintaining constant muscle length is suggested to be based on complex control processes, it is assumed that the AFiso_max_ might be more vulnerable in reaction to probable influencing factors such as disgusting odours than the AFecc_max_, MVIC or the commonly assessed eccentric or concentric strength.

The aim of this pilot study was to investigate whether the AF in healthy participants shows different patterns in reaction to neutral, pleasant and disgusting odours. Since unpleasant odours (disgust) could function as a threatening stimuli which may elicit an emotionally related inhibition of the muscular activity, we hypothesized that disgusting odour will reduce the maximal holding capacity (AFiso_max_). We expected no effect of disgusting odours on the maximal eccentric AF (AFecc_max_). Pleasant or neutral odours were assumed to have no reducing effect neither on the AFiso_max_ nor on the AFecc_max_.

## Methods

### Participants

The Adaptive Force (AF) of the hip or elbow flexors of n = 10 healthy participants was recorded by a handheld device during the manual muscle test (MMT) performed by two experienced testers (tester 1: female, 34 years, 168 cm, 55 kg; 8 years of MMT experience; tester 2: male, 63 years, 185 cm, 87 kg; 25 years of MMT experience). The anthropometric data of the healthy participants are given in Table 1 (detailed information are given in supplementary material Table S1). Exclusion criteria were any kind of health issues and an impaired neuromuscular function of the tested muscles assessed by the MMT prior to the measurements.

**Table 1:** Anthropometric data. Displayed are the arithmetic means and standard deviations (M ± SD) of age, height and body mass of the n = 10 participants.

Gender	Age (years)	Height (cm)	Body mass (kg)
Female (n = 6)	36.00 ± 14.71	162.60 ± 7.96	65.20 ± 10.18
Male (n = 4)	31.75 ± 8.54	186.00 ± 2.45	79.00 ± 7.44

The study was conducted according to the guidelines of the Declaration of Helsinki and approved by the Ethics Committee of the University of Potsdam, Germany (protocol code 35/2018; 17.10.2018).

### Handheld device for recording the dynamics and kinematics during the manual assessment of Adaptive Force

The handheld device (development was funded by the Federal Ministry of economy Affairs and Energy; project no. ZF4526901TS7) consists of strain gauges (co. Sourcing map, model: a14071900ux0076, precision: 1.0 ± 0.1%, sensitivity: 0.3 mV/V) and kinematic sensor technology (Bosch BNO055, 9-axis absolute orientation sensor, sensitivity: ± 1%) to record the reaction force, the accelerations and angular velocity (gyrometry) between tester and participant during the MMT. All data were buffered with a sampling rate of 180 Hz. The data were AD converted and sent via Bluetooth 5.0 to a notebook. A measuring software (based on National Instruments Lab-view) saved the transmitted data.

### Manual muscle testing

The manual muscle test (MMT) is a clinical method of testing the AF as a marker of neuromuscular functioning^49^. For the present investigation, the so-called “break test” was applied^39,49^, which is usually conducted in submaximal intensities. Thereby, the tester applies an increasing force by pushing against the subject’s limb, whereby “the subject is asked to resist the tester’s gradually increasing pressure”^49^. On the tester’s side the maximally producible force is limited by the prescribed positioning to execute the test. The muscular strength of the tester would easily allow to generate a higher force, but it would lead to a displacement of the tester’s stance. Of course, individual anthropometric properties are additional factors which influence the maximal applicable force, but the testing position is the crucial limit. In preceding measurements, the two involved testers developed maximum forces around 280 N. With this amount one would normally not be able to overcome a well-functioning hip or elbow flexor group of young healthy subjects. Therefore, the MMT is not designed to measure the subject’s maximal strength. (This could be done using technical devices which could easily overwhelm the subject.) The aim of the MMT is to check on how good the neuromuscular system of the participant is able to adapt to an external force increase. Hence, the MMT evaluates the submaximal stability of the muscle with respect to an increasing external force. On this account, the referred AFmax does not represent the subject’s maximal strength, but the force which is maximally applied to the subject during interaction. If the adaptation during the force increase is optimal, the muscle length will stay quasi-isometric during the whole test until an oscillating force equilibrium on a considerably high force level is perceived by the tester^39,49^. In case of a failing adaptation, the muscle would already start to lengthen (breaking point) during the force increase clearly below the level of maximal voluntary isometric contraction (MVIC). Therefore, the assessment of the MMT by the tester is differentiated into two conditions^39,49^: the MMT is assessed as “stable” in case the limb of the subject maintains the isometric position though the whole force increase. An “unstable” MMT is classified if the limb gives way during the force rise on a submaximal force level.

Because of this manual approach, the test and its interpretation are subjective. By using the newly developed handheld device the force profile and the position of the tested limb can be objectified simultaneously during the MMT by recording the dynamics and kinematics. A recent investigation showed that experienced testers are able to reproduce the force application in a reliable way (see below), which is one prerequisite for the present investigation^39^.

### Characteristic and reproducibility of force profile

**Figure 1:**
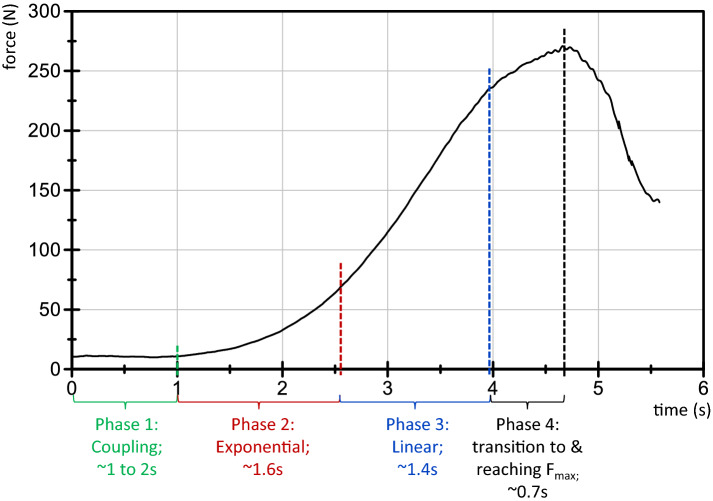
Schematic force profile. The force increase applied externally by the tester during the MMT consists of the four illustrated phases. (according to Bittmann et al.^39^).

The applied force profile during the MMT was defined to have the progression as displayed in Fig. 1^39^. During phase 1, the tester and the subject get in contact on a low force level for 1–2 s. This is necessary to create a starting force level, so that the subject gets the opportunity to adapt to the tester’s external force application at all (initially on a consistent low force level). In phase 2, the tester increases the force smoothly in an exponential way. At the beginning, small steps of force rise are necessary, so that the subject gets a chance to adapt to the increasing force (for neurophysiological explanation see^39^). This second exponential phase merges into a linear force rise in phase 3. If an oscillating force equilibrium between tester and subject is reached, this should be maintained for a few seconds, whereby the maximal AF of the test is reached (phase 4). Then the tester stops the interaction and the force decreases again. The duration of this whole force rise (phase 2 to 4) up to the maximal reaction force should be within 4 s. Of course, this force application depends especially on the tester. A sufficient reproducibility of the applied force profile is a necessary precondition for valid data.

**Figure 2:**
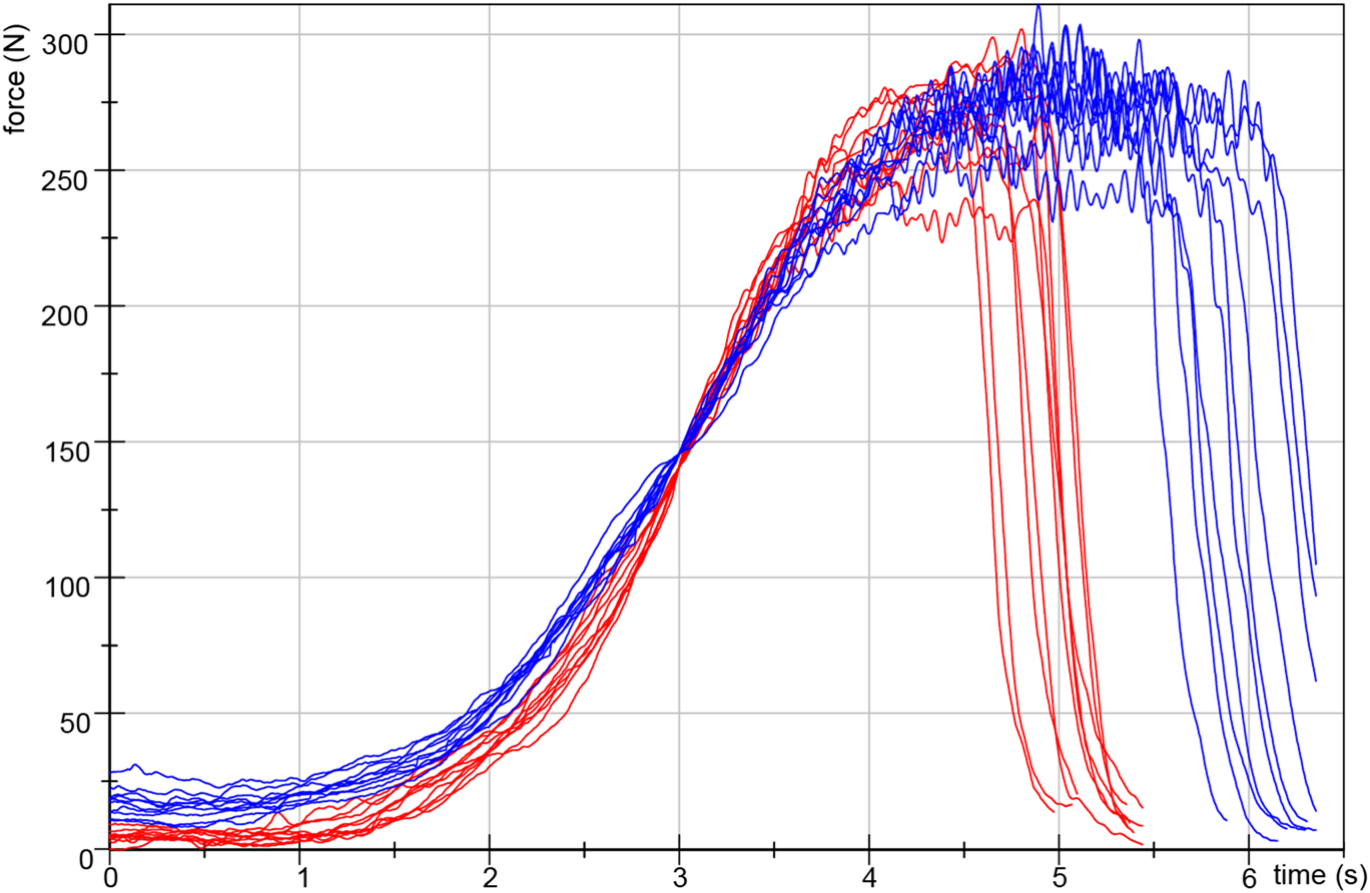
Repeated force profiles against a stable resistance. Ten repeated force profiles of tester 1 (female, red) and tester 2 (male, blue) against a stable resistance in the MMT setting of the hip flexors (filtered with Butterworth, cut-off frequency 20 Hz, filter degree 5). (according to Bittmann et al.^39^).

Prior to the study, both testers proofed their ability to test in a reproducible way by performing 10 repeated force increases against a stable resistance in the MMT setting of the hip flexors. The setting was equivalent to the here performed one (see below), except for a fixed leg of the participant to exclude its reaction as a second influencing factor^39^. The force profiles of both testers are illustrated in Fig. 2. The coefficient of variation of the maximum force amounted 5.6% (tester 1) and 4.6% (tester 2), respectively. Both testers showed a reliable slope from start to maximum force comparing the 10 trials with an intraclass correlation coefficient of ICC(3,1) = 0.992 (tester 1) and 0.995 (tester 2), respectively. Furthermore, the inter-tester reproducibility of both testers is considered as high with ICC(3,1) = 0.989 and Cronbach’s alpha of 1.0. Therefore, the force profiles of the two experienced testers, which performed the MMT here, can be considered as reliable. Group comparisons between experienced, little experienced and unexperienced testers showed significant differences in several parameters of force profile in a previous study^39^.

### Setting and procedure

Prior to the measurements, the participant was introduced to the procedure and gave its written consent to participate. Subsequently, the MMT of hip flexors was executed preliminarily. In case of full stability, those were chosen for the investigation. If it did not fulfil this requirement, the elbow flexors were used provided the MMT was fully stable. Afterwards, the participants selected the most pleasant and most disgusting odours out of 12 standardized sniffing sticks (olfasense GmbH) (rating − 5 or + 5 on a scale of − 5 (disgust) to + 5 (pleasant)). Those sniffing sticks are normally used for testing the olfactory capacity in clinical circumstances, e.g., in Parkinson’s disease. After a break for neutralization of the olfactory sense, the AF of the participant was tested by the MMT performed by the same tester with the handheld device while smelling at different odours (single-blinded, randomized): 3 × neutral, 3 × pleasant, 3 × disgusting (in some subjects less than three trials per odour were performed; 1 trial had to be excluded because of technical problems; for further information see supplementary material Table S2). A double-blinded study is not possible since the participant will always smell the odour. However, the participants were instructed to not show any reaction with respect to the odours, so that the tester was not influenced by possible hints on which odour was presented. The order of stick presentation was randomized. An assistant gave the sticks to the participant and recorded the measurements. Tester 1 tested n = 6 subjects (1 × elbow flexors, 5 × hip flexors), tester 2 tested n = 4 subjects (1 × elbow flexors, 3 × hip flexors) (for further information see supplementary material Table S2).

**Figure 3:**
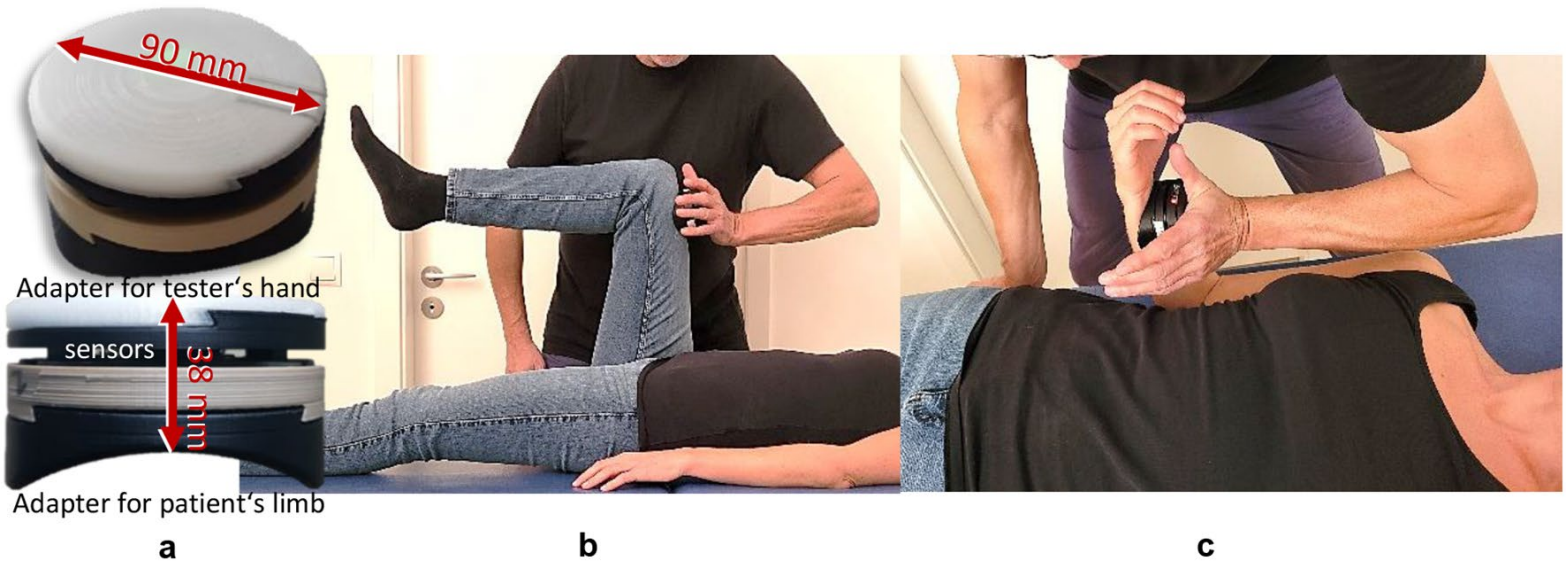
Setting of the manual AF measurements. The handheld device (**a**) is placed between the palm of the tester and the limb of the participant; (**b**) manual muscle test (MMT) of the hip flexors; (**c**) MMT of the elbow flexors. The sniffing stick (olfasense) is held by the participant to his or her nose to smell the odour during the MMT.

The MMT was performed in the following way: the subject lay in supine position with a hip and knee angle of 90° for testing the hip flexors (Fig. 3b). The tester had contact to the distal end of the thigh of the participant. The handheld device (Fig. 3a) was located between the tester’s palm and the participant’s thigh to measure the dynamics and kinematics during the MMT. For testing the elbow flexors, the participant was supine and flexed its elbow joint in 90° with a maximal supination (Fig. 3c). The tester had contact with the handheld device at the distal forearm of the participant. In both settings, the exact placement of the device at the respective limb was marked to reproduce the position during subsequent measurements. The force rise was applied by the tester in direction of muscle lengthening of the participant’s hip flexors (hip extension) or elbow flexors (elbow extension), respectively.

The task of the participant was to maintain (hold isometrically) the respective starting position for as long as possible while adapting to the external force rise applied by the tester. The handheld device detected the limb position during the force rise. After the test, the tester gave his or her judgement regarding the subjectively felt stability during the test. In case, the position of the limb stayed stable by maintaining the same muscle length and joint angle during the whole duration of force rise the MMT was assessed as “stable”. If the participant merged into eccentric muscle action in the course of force increase the MMT was rated as “unstable”.

### Data processing and statistical analysis

For evaluation, the force and gyrometer signals were used. The csv-files were transferred to DIAdem 2017 (National Instruments). All signals were interpolated (linear spline interpolation) to ensure equidistant time channels (sampling rate: 1000 Hz) and filtered (Butterworth, cut-off frequency 20 Hz, filter degree 5; for slope parameter to eliminate the oscillations: cut-off frequency 3 Hz, filter degree 10). The parameters of interest are the following:

#### The maximal Adaptive Force (AFmax)

This parameter refers to the maximal reached force value during the whole trial. AFmax (N) can be reached under two different circumstances. If the muscle length stayed stable over the whole force rise, AFmax was reached under isometric conditions (AFiso_max_). In case of yielding during force increase, this value was obtained during eccentric muscle action (AFecc_max_). The AFmax does not necessarily reflect the maximal strength of the participant, since it depends on the amount of force applicated by the tester (see above).

#### The maximal isometric Adaptive Force (AFiso_max_)

This parameter refers to the maximal reached AF during holding isometric muscle action; thus no muscle lengthening occurred until this moment. The gyrometer signal was used to determine the breaking point indicating the starting of muscle lengthening. It oscillates around zero under isometric circumstances. In case the muscle lengthened the gyrometer signal decreased below zero. The force value at the moment of last zero crossing of the gyrometer signal was defined as AFiso_max_ (N) indicating a deviation of the angle over time. In case the muscle did not lengthen, objectified by a gyrometer signal oscillating around zero over the whole MMT, the maximum force value AFmax = AFiso_max_. The parameters AFiso_max_ and the ratio of AFiso_max_ to AFmax (%) were used for further considerations.

#### The Adaptive Force in the moment of onset of oscillations (AFosc)

The force signal showed an onset of oscillation in the course of force rise in some trials. Therefore, the oscillations of force signal were evaluated in NI DIAdem 2017. If three consecutive maxima with a time distance of  < 0.15 s were identified, this was defined as the onset of oscillations. The border of 0.15 s was set since muscular oscillations occur in a low frequency range of ~ 10 Hz^50–53^. The AF value at the first of those three oscillations referred to AFosc (N). If no onset of oscillation was present, AFmax = AFosc. The parameter AFosc and the ratio of AFosc to AFmax (%) were used for further considerations.

#### Slope of force rise

This parameter was evaluated to ensure the reproducibility of force rises comparing the trials with neutral, pleasant and disgusting odours. According to neurophysiological considerations and empirical experience^39^, the characteristic of the force rise might affect the outcome. To compare the slopes of the trials with neutral, pleasant and disgusting odours until the breaking point, the slope in phase 3 was calculated with reference to the breaking point in trials with unstable condition. For that, the arithmetic mean of the AFiso_max_ values of the “unstable” trials was used as reference for each participant (AFiso_unst_). The slope of force curve was then calculated by the difference quotient from the time and force values at 60% of AFiso_unst_ to 100% of AFiso_unst_ for each trial and participant. Due to the exponential force rise, the decadic logarithm was taken from the slope values. The logarithmized slope is given by lg(N/s). In five as stable assessed trials, the 100% of AFiso_unst_ was reached in the transition to phase 4. To avoid a distortion of slope results those trials were excluded from the slope analysis.

The arithmetic means (M), standard deviations (SD) and 95%-confidence intervals (CI) of all parameters were calculated per participant separately for trials with neutral, pleasant and disgusting odours. All parameters were statistically compared between the three odours using IBM SPSS Statistics 27 to identify possible differences between the odours. For that, the normal distribution was checked with the Shapiro–Wilk test. In case normal distribution was not fulfilled, the Friedman test was used. This was the case for both ratios ($\frac{{{\text{AFiso}}_{\max } }}{{{\text{AF}}_{\max } }}$; $\frac{{{\text{AFosc}}}}{{{\text{AF}}_{\max } }}$). All other parameters were normally distributed and the ANOVA for repeated measurements was performed (RM ANOVA). In case the sphericity was not fulfilled (Mauchly test), the Greenhouse–Geisser correction was applied. For post hoc test, Bonferroni correction was applied. The effect size eta squared (η^2^) was calculated by SPSS. For pairwise comparisons between the odours, the effect size Pearson’s r was calculated by $r = \left| {\sqrt {\frac{{t^{2} }}{{t^{2} + df}}} } \right|$ for RM ANOVA and by r = $\left| {\frac{z}{\sqrt n }} \right|$ for Friedman test. Significance level was set at α = 0.05.

### Informed consent

Informed consent was obtained from all subjects involved in the study.

## Results

**Figure 4:**
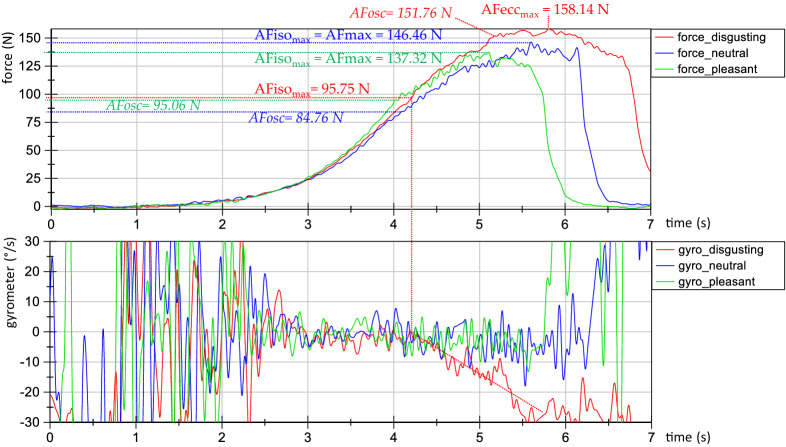
Exemplary signals. Displayed are the force (N) (above) and gyrometer signals (°/s) (below) during manual muscle test (MMT) of tester 1 testing the same female participant (age: 44 years, height: 173 cm, body mass: 77 kg) during disgusting (red), neutral (blue) and pleasant (green) odours. Marked are the parameters AFmax, AFecc_max_, AFiso_max_ and AFosc (according to Figure 12 in ^39^).

Exemplary force and gyrometer signals during the MMT of the hip flexors of one female participant during neutral, pleasant and disgusting odours are displayed in Fig. 4. As can be seen, the force rises were nearly identical for all three trials, especially in the first three phases (Fig. 4 above). This illustrates the high reliability of the tester’s force application during the MMTs. Furthermore, the gyrometer signal (Fig. 4, below) of disgusting odour decreased clearly, whereas during neutral (blue) and pleasant (green) odours, the gyrometer signals stayed stable, oscillating around zero (defined as isometric behaviour) until the maximal AF (AFmax was reached. Thus, the values of AFmax correspond to AFiso_max_ during neutral and pleasant odours. During disgusting odour the AFiso_max_ = 95.75 N. This amounts 60.5% of the AFecc_max_ = 158.14 N, which was reached under muscle lengthening. The participant started to lengthen her muscle at a considerably lower force level (AFiso_max_) during disgusting odour, whereby a muscle lengthening did not occur during neutral or pleasant odours. It is important to mention that the AFiso_max_ during disgusting odour arose at a 35% and 30% lower force level compared to the AFiso_max_ during neutral and pleasant odours, respectively. During muscle lengthening (disgusting odour), the participant was able to produce an even slightly higher maximal force compared to the AFmax during neutral and pleasant odours. However, during neutral and pleasant odours, the AFmax of the test was reached under isometric conditions (AFiso_max_) and under the appearance of oscillations. The oscillations during disgusting odour appeared at a force level of AFosc = 151.76 N, which amounts to 96% of the corresponding AFecc_max_. The onset of oscillations during neutral and pleasant odours occured at a force level of AFosc = 84.76 N and AFosc = 95.06 N, respectively, which amounts 58% and 69% of the related AFiso_max_ (Fig. 4). The AFosc during neutral and pleasant odours amounted to 56% and 63% of the AFosc during disgusting odour and appeared at a lower force level than the AFiso_max_ during disgusting odour.

This example illustrates the behavior of AF during different odours, which consistently appears in 73 of all 76 measurements (the three exceptions are described below). This is supported by the following statistical group comparisons (Table 2).

**Table 2:** Displayed are the arithmetic means (M), standard deviations (SD), lower and upper border of 95%-confidence intervals (CI) as well as the p-values and effect sizes η^2^ of all parameters comparing the groups neutral, pleasant and disgusting odours.

Parameter	odour	M ± SD	CI	Significance p	η^2^
AFmax (N)	Neutral	189.58 ± 40.63	164.38; 214.75	0.051	0.334^1^
	Pleasant	201.70 ± 37.14	178.64; 224.72		
	Disgusting	219.85 ± 35.19	198.04; 241.66		
AFiso_max_ (N)	Neutral	185.95 ± 40.80	160.66; 211.23	**< 0.001**	0.698^1^
	Pleasant	197.32 ± 34.52	175.92; 218.71		
	Disgusting	131.46 ± 30.03	112.85; 150.07		
Ratio AFiso_max_ to AFmax (%)	Neutral	98.39 ± 3.39	96.29; 100.49	**< 0.001**^2^	–
	Pleasant	98.04 ± 2.33	96.59; 99.48		
	Disgusting	59.88 ± 10.26	53.52; 66.24		
AFosc (N)	Neutral	145.70 ± 46.76	116.71; 174.68	**< 0.001**	0.690^1^
	Pleasant	155.09 ± 31.87	135.34; 174.85		
	Disgusting	206.67 ± 38.03	183.10; 230.23		
Ratio AFosc to AFmax (%)	Neutral	75.22 ± 10.22	68.89; 81.55	**0.001**^2^	–
	Pleasant	77.04 ± 8.73	71.63; 82.45		
	Disgusting	93.86 ± 7.16	89.43; 98.30		
Slope lg(N/s)	Neutral	1.97 ± 0.13	1.89; 2.06	0.485	0.098^1^
	Pleasant	1.99 ± 0.09	1.94; 2.05		
	Disgusting	1.96 ± 0.12	1.89; 2.03		

^1^Eta squared η^2^ of RM ANOVA; ^2^Friedman test. Significant results are displayed in bold.

### Assessment of the manual muscle test by the tester

In total, 24 of 25 trials with neutral odour were rated as “stable” by the testers. One trial was assessed as “unstable” by tester 1. With pleasant odour, 25 of in total 26 trials were assessed as “stable” and one as “unstable” (tester 2), whereby the participant reported “sensing” her groin (no pain, appeared in no further measurement). 24 of in total 25 trials with disgusting odour were assessed as “unstable”, one trial was assessed as “stable” (tester 1). (For detailed information see supplementary material Table S2). Regardless of the testers’ subjective assessments, the following evaluation is only based on the grouping related to the presented odours.

### Slope of force profiles

**Figure 5:**
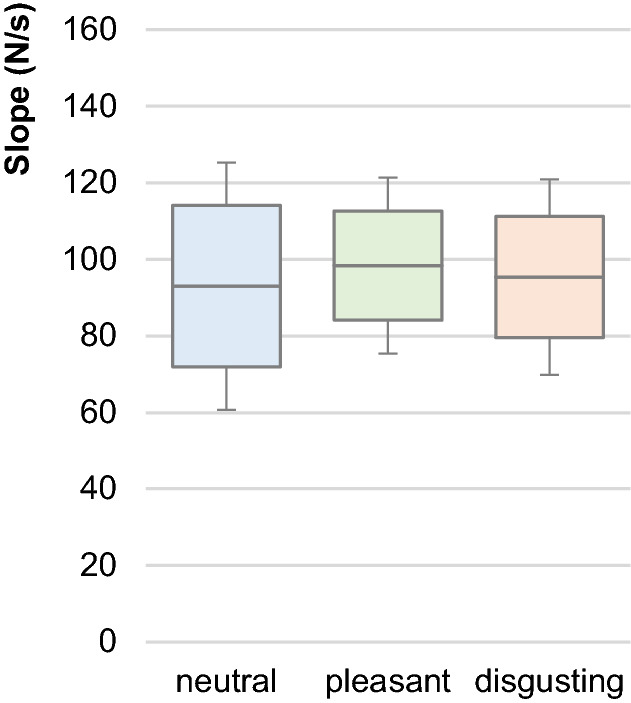
Slope. Displayed are the arithmetic means, standard deviations (error bars) and the 95%-CIs of the decadic logarithmus of slope from 60 to 100% of AFiso_unst_ lg(N/s) comparing the different odours neutral (blue), pleasant (green) and disgusting (orange). The statistical comparisons turned out to be non-significant (p > 0.05).

The slope is the main parameter to investigate the reproducibility of the testers’ force rises. As can be seen in Table 2, Figs. 4 and 5, the slopes did not differ significantly between neutral, pleasant and disgusting odours (F(2,14) = 0.762, p = 0.485). Therefore, the following considerations of AF parameters are based on the requirement of reproducible force profiles between the MMTs during perception of the three odours.

### Maximal Adaptive Force and maximal isometric Adaptive Force

**Figure 6:**
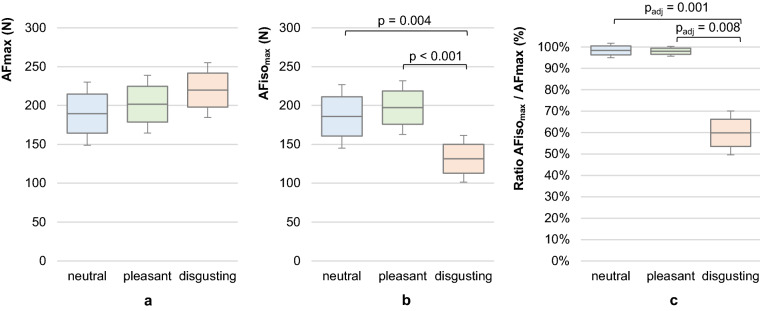
Maximal Adaptive Force and maximal isometric Adaptive Force. Displayed are the arithmetic means, standard deviations (error bars) and 95%-CIs of (**a**) the maximal Adaptive Force (AFmax), (**b**) the maximal isometric Adaptive Force (AFiso_max_) and (**c**) the ratio of AFiso_max_ to AFmax comparing the odours neutral (blue), pleasant (green) and disgusting (orange). The p-values of significant comparisons are given.

The overall maximal AF (AFmax), which occurred under isometric or eccentric muscle conditions, is slightly but not significantly higher in measurements with disgusting odours compared to neutral (p = 0.050) or pleasant odours (p = 0.086), respectively (Fig. 6a). The AFmax during neutral odour amounted averagely 93.95 ± 10.35% of the AFmax during pleasant odours and 87.20 ± 17.76% of the AFmax during disgusting odours. The AFmax during pleasant odours amounted averagely 92.28 ± 12.97% of the AFmax during disgusting odours. The maximal isometric AF (AFiso_max_) was significantly lower in measurements with disgusting odours (131.46 ± 30.03 N) compared to neutral (185.95 ± 40.80 N; p = 0.004, r = 0.79) and pleasant odours (197.32 ± 34.52; p < 0.001, r = 0.89), respectively (F_Green_ (1.285, 11.536) = 20.790, p < 0.001) (Table 2, Fig. 6b). The AFiso_max_ did not differ significantly between neutral and pleasant odours (p = 0.105, r = 0.52). In the trials with disgusting odours, the AFiso_max_ amounted averagely 60 ± 10% of the related AFmax, whereas with neutral or pleasant odours, the ratio was significantly higher with 98 ± 3% (p_adj_ = 0.001, r = 0.52) and 98 ± 2% (p_adj_ = 0.008, r = 0.43), respectively (Table 2, Fig. 6c). Furthermore, the AFiso_max_ during disgusting odours was averagely 73.23 ± 12.06% of the AFiso_max_ with neutral and 67.62 ± 15.85% of the AFiso_max_ with pleasant odours, respectively. That indicates that during perception of a disgusting odour, the maximal isometric AF was significantly lower compared to neutral or pleasant odours. The participant was not able to appropriately resist the external increasing force in an isometric way under the perception of a disgusting odour; the muscle started to lengthen at a substantially and significantly lower force level compared to neutral or pleasant odours, whereby the AFmax was statistically similar between all odours.

### Adaptive Force at the onset of oscillations

**Figure 7:**
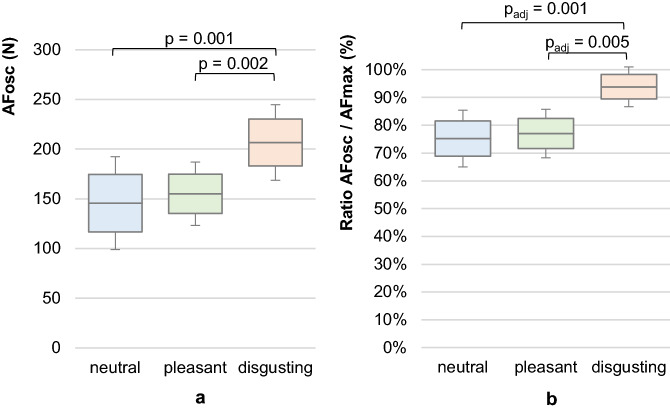
Adaptive Force at onset of oscillations. Displayed are the arithmetic means, standard deviations (error bars) and 95%-CIs of (**a**) the Adaptive Force at the moment of onset of oscillations (AFosc) and (**b**) the ratio of AFosc to AFmax comparing the different odours neutral (blue), pleasant (green) and disgusting (orange). The p-values of significant comparisons are given.

The measurements with neutral or pleasant odours were characterized by an onset of oscillations during the force rise at a submaximal force level. Those oscillations did not or only slightly occur at a high force level during perception of a disgusting odour. Significant differences with p < 0.001 arose comparing AFosc between neutral, pleasant and disgusting odours (Table 2). The pairwise comparisons revealed a significant difference between disgusting and neural odours (t(9) = − 4.952, p = 0.001, r = 0.86) and between disgusting and pleasant odours (t(9) = − 4.432, p = 0.002, r = 0.83) (Fig. 7a). The AFosc did not differ significantly between neutral and pleasant odours (t(9) = − 1.579, p = 0.149, r = 0.47).

Looking at the ratio of AFosc to AFmax, the significant difference was confirmed by the Friedman test (χ^2^(9) = 15.20, p = 0.001). The Bonferroni post-hoc test revealed p-values of p_adj_ = 0.001 comparing neutral and disgusting odours (z = − 3.578, r = 0.51) and p_adj_ = 0.005 for pleasant and disgusting odours (z = − 3.130, r = 0.44) (Fig. 7b).

## Discussion

The presented study investigated the dynamics and kinematics during the manually tested AF utilizing a handheld device in healthy participants under the influence of neutral, pleasant or disgusting odours. The evaluation of the slope of force rises revealed a non-significant difference between the three odours. Accordingly, the following discussion is based on reliable force applications of the testers. The main outcomes are:

The maximum AF (AFmax) did not differ significantly between the three odours. The main difference was that by smelling neutral and pleasant odours, the AFmax was reached under isometric conditions (AFiso_max_); whereas by perceiving disgusting odours, the AFmax was obtained during muscle lengthening (AFecc_max_). The AFiso_max_ was significantly lower under the influence of disgusting compared to pleasant and neutral odours. This indicates that during disgusting odours, the participants merged into eccentric muscle action at a significantly lower force level (~ 60% of AFecc_max_), whereas under neutral or pleasant olfactory influence, isometric stability was maintained almost until the maximum. That confirms the hypothesis that the maximal isometric AF, but not the AFmax, decreases during perception of a disgusting odour.

The AF at the oscillation onset was significantly lower for neutral and pleasant compared to disgusting odours, in which no or only poor oscillations occurred at a high force level. This indicates that the AF in healthy persons perceiving neutral or pleasant odours is characterized by oscillations, which emerged during the force rise at ~ 75% of the maximal force level.

### Limitations

The testers’ force profile application might be the main limitation in this investigation. As mentioned above, the force application must be reproducible and appropriate as suggested in^39^. A smooth start followed by a faster linear force increase might be suitable to test the adaptive capacity of the neuromuscular system^39^. The testers proofed their ability to test reproducibly prior to the investigation and the slope was used as parameter to control the force increase. The slopes did not differ significantly in the present measurements between the MMTs with different odours. The slope prior to the breaking point is even slightly lower in measurement with disgusting compared to pleasant odours (− 3%). This speaks against the frequently appearing criticism that an unstable MMT is due to a steeper force rise. Nevertheless, the slope might be one crucial parameter when applying the force rise and must be controlled. An assessment of the force application by recording the dynamics and kinematics during MMT should take place to verify reliable and valid results.

Furthermore, the reached maximal force of a stable muscle depends not only on the participant, but also on the tester. The force profile is a result of their interaction. Because the participant was only reacting in a holding way, the tester determined the course of force including its maximum when a stable muscle is tested. That is why it depended on the tester to what extend the participant’s holding capacity was challenged under stable conditions. Due to biomechanical aspects, it is mostly not possible to overcome the here tested hip and elbow flexors. However, in general, if the tester applies a lower maximal force, the participant’s response will naturally be lower, too. Therefore, the AFmax does not reflect the real maximal strength of the participant, since it depends on the amount of force applicated by the tester. As mentioned above, the “break test” is characterized by a force application in submaximal areas. However, the AFiso_max_ under unstable conditions will refer to the maximal holding AF under the obviously impairing influence of a disgusting odour. The AFecc_max_ monitors the maximal eccentric force of the participants under the given circumstances. Since the AFiso_max_ under stable conditions and the AFecc_max_ under unstable conditions differed not significantly in the present study, it is assumable the applied force of the testers was close to the maximal force capacity of the participants; with the assumption that the AFecc_max_ is not changed by the influence of disgusting odors. Since the MMT was performed in submaximal areas, no statement can be made regarding the behavior of AFecc_max_ under the effect of neutral or pleasant odours.

A tendency of a lower AFmax was visible for the tests under stable (neutral/pleasant) compared to unstable conditions (disgusting) (Fig. 6A). This could be comprehended as a possible reason for the different muscle states. However, the decisive difference is that the breaking point (AFiso_max_) in unstable conditions (disgusting) appeared at a substantially and significantly lower level compared to the maximal force which the muscle reached under stable conditions (neutral/pleasant) without muscle lengthening.

Another limitation is the small sample size (n = 10). However, the significances and effect sizes are considerably high. That is why we regard these preliminary results as a valuable first consideration reflecting the neuromuscular control of healthy subjects in reaction to olfactory stimuli. The sample size must, of course, be increased to verify the found results.

Eventually, there could be a concern regarding a possible confounding factor. Although the subjects were instructed to show no verbal or nonverbal reaction to the exposed odours and the testers avoided to get into visual contact with the subject prior to and during the MMTs an unconscious influence cannot be ruled out completely. In this case, the tester could involuntarily have changed his or her profile of force application and, therefore, influenced the outcome. An unaware sudden start and steeper course of force rise would have favoured an unstable behaviour of the tested muscle. This is one reason why the slope before the breaking point was considered. The results invalidate the concern about unconscious manipulations by the tester because there is no relevant difference of slope between the odours.

### Characterization of “stable” and “unstable” adaptation

Taken the above-mentioned results together, it is suggested to define a “stable” and an “unstable” adaptation to an increasing external force as follows. A stable adaptation can be characterized by two conditions: (1) the AFiso_max_ ≈ AFmax (≥ 98% of AFmax), thus, the muscle length stays quasi-isometric during the whole force rise (slight muscle suspensions are acceptable); (2) Oscillations of force with about 10 Hz arise during force increase, thus, AFosc is significantly lower than AFmax. Based on the data, a percentage of averagely 76 ± 9% of AFosc to AFmax can be expected. An unstable adaptation is characterized by the following two conditions: (1) AFiso_max_ is considerably lower than AFmax. Thus, the muscle lengthens during the force rise in submaximal areas and the maximum is reached under eccentric conditions (AFecc_max_). Based on the data, a percentage of 60 ± 10% of AFiso_max_ to AFecc_max_ can be expected. (2) No or only poor oscillations on a high force level occur during the force rise, thus, AFosc is close to AFecc_max_ with a ratio of 94 ± 7%.

It is suggested that the unstable behaviour reflects an inadequate adaptation of muscle length and tension to external increasing force applications. In the present study, this emerged by presenting a disgusting odour. This obviously is impairing the muscle function in the sense of AF in the here investigated small sample size of 10 healthy participants. For a first cautious summary thereof, a well-functioning undisturbed neuromuscular adaptation to an external force increase seems to be characterized by a sufficiently adapted muscle tension maintaining muscle length and limb position as well as by the occurrence of mechanical oscillations.

### Neurophysiological explanation of muscular adaptations with regard to perception of olfactory inputs

Based on the own research, there are no comparable investigations concerning the behaviour of AF—or other force outputs of limb muscles—in reaction to different odours. Trying to understand the underlying mechanisms, the suggestion of neuromuscular AF processing should be regarded in more detail. During the manual assessment of AF, the tested participant receives sensory inputs due to the tester’s contact and force application. Hereby, skin and joint receptors, muscle spindle cells and Golgi tendon organs are perceiving mechanical inputs. The sensory signals are forwarded through the posterior horn to other spinal and supraspinal structures^54–56^ and provide the current state of muscle length, tension and joint position. Sighting the literature, one can assume that at least the thalamus, cerebellum, inferior olivary nucleus (ION), red nucleus, basal ganglia, cingulate cortex and the sensorimotor cortices are involved in the complex processing of adaptive motor control and are interconnected directly or indirectly^7,36,37,42,54–91^. The cerebellum is considered as one of the most relevant sensorimotor structures concerning the temporal-spatial processing^59,67^. Its anterior part seems to be especially relevant for sensorimotor functions and the posterior part for cognition and emotions^72^. However, the posterior cerebellum also seems to be involved in the “prediction of sensory events”, especially for “timing perception and adjustment”^67^. Therefore, the cerebellum is relevant regarding the motoric adaptation^42,73^, whereby it appears to be of particular importance at the beginning of an adaptation^73^. As mentioned in the introduction, a mixed mechanism of feedback and feedforward control is assumed to be involved in the adaptive process^42^. The cerebellum seems to work as the forward controller in cooperation with the ION, which provides the motoric time signal^57–60^. Thereby, the cerebellum can learn to predict the accurate timing of connected events and, thereby, intervenes in motor control^57,58,84^. This flows into the error processing of motor control and provides the rhythmic neuronal signal to enable temporal coordinated movements^57–59^. The cerebellum receives information of the muscle spindle, Golgi tendon organs and skin receptors^54^. Therefore, it might be essential for the target-actual comparison of muscle length and tension. Reafferences are compared with a copy of the initial motoric command^36^. Mismatches are then corrected by adjustments of the motor output. It was suggested that the cerebellum is a kind of “error-correcting machine”, which compares the “expected and actual outcome of a sensory prediction or motor command”^63^. Also, other central structures seem to be relevant thereby. The parietal cortex was suggested as a central interface between sensory and motor processes concerning temporal processing^83^. Additionally, the thalamus is a central switching point for sensory and motor processes^76^, with its main task of modulating and regulating the flow of information to the cortex^77^. Meanwhile, the involvement of the cingulate cortex in emotions, pain processing as well as in spatial and motor control is secured^7,37,66,80^. This area reacts to different sensory inputs, e.g., exteroception, proprioception and nociception, and has a wide interconnection to other central structures^7,66^. Additionally, the basal ganglia work as a kind of filtration station for the muscle tone, including temporal processing, by facilitating desirable and inhibiting undesired motoric programs^37,64,92^. Furthermore, the limbic system, especially the amygdala^8^ and the hippocampus^48^, are involved in motor control and in the processing of olfaction and emotion^8,48^. Last but not least the motor cortex receives information of the thalamus, the cerebellum, the basal ganglia, the red nucleus and of the limbic system^67,70^. The premotor cortex as well as the supplementary motor area of the cerebral cortex are involved in the temporal processing of motor activity^83,85,93^. Therefore, all those networked structures seem to be relevant in controlling the muscle length and tension during adaptation to external forces. Jörntell suggests, that “the final motor command, i.e. the final spatiotemporal structure of the activation of the α-motoneurons and thereby the muscles, is a sum or a product of all the motor command signals issued and the pattern of sensory feedback”^65^.

As mentioned above, the occurred oscillations of ~ 10 Hz during stable conditions (neutral/pleasant odours) might indicate a relevance of mechanical muscle oscillations in interaction with external forces. Those did not or only sparsely arise during unstable muscle states (disgusting odours). Oscillations are also found in the mechanical^50–53^ as well as in electrical muscular activity^88,94–98^ during isometric muscle actions. They also occur in central structures during muscular activity. The cerebellum shows great inhibitory postsynaptic potentials of ~ 8–17 Hz, which were found in cats^62^. Also other vertebrates exhibit oscillatory activity of the olivocerebellar circuitry of 10 Hz^62,86^. Additionally, the thalamus and neurons of the motor cortex are characterized by discharge frequencies between 11–30 Hz^76^ and ~ 10 Hz^99–101^, respectively. Furthermore, the long latency reflexes of proprioception are processed with ~ 10 Hz^81,87^. If an external force is changing, the corresponding correction also takes place with latencies of ~ 10–12 Hz^56,90^. It is hypothesized that the found oscillations during the MMT could represent the normal functioning of the complex neuronal network standing behind it. With this prospect, their absence could possibly indicate irritations.

If the regulative circuitries are working properly, the adaptation in the sense of AF ought to be performed adequately (“stable”). The neuromuscular system should be able to adapt appropriately to the external force increase in time and space if the force increases not too abrupt or intense. However, the present study showed that this neuromuscular adaptation might be impaired by perceiving disgusting odours. Olfactory stimuli are processed by different regions as mentioned above. The piriform cortex and the limbic system (amygdala, hippocampus)^5,6^ have to be highlighted because of their close connection to olfaction, emotions and motor control^45–47,102,103^. Especially with perception of pleasant or disgusting odours, we assume the occurrence of positive or negative emotions, respectively, as was suggested by several authors^1,8,9,16^. Therefore, it is likely that the here found reduced AFiso_max_ and later occurred AFosc at a higher force level during perception of a disgusting odour might be related to a negative emotional component. A pilot-study investigating the AF under the influence of different emotionally related imaginations was performed and the results will be presented soon.

### Characterization and specialty of the isometric Adaptive Force

The results strongly indicate that under particular circumstances a muscle can yield in length at a substantial submaximal force level. In this case, the muscle looses its stability (ability to hold) despite of its further increase of tension. The maximal holding capacity (AFiso_max_) changed within a few seconds depending on the olfactory stimuli. Therefore, in contrast to AFecc_max_, AFiso_max_ seems to be sensitive regarding disgusting olfactory inputs, which are interpreted as possible disturbing factors. The arising oscillations  ~ 10 Hz under stable conditions suggest this could not only be a characteristic of maintaining muscular stability but probably a prerequisite. A loss of this function could be a sign of a disturbed sensorimotor processing characterized by a muscle lengthening at a considerably low AF. It is suggested that the AFiso_max_—presumably depending on the onset of oscillations—seems to be the most vulnerable and, therefore, the probably most relevant parameter in adapting to external forces. The immediate response of AFiso_max_ to the here investigated olfactory inputs strongly indicate that it is based on regulatory mechanisms. Because of the close linkage of olfactory and emotional processing, the observed effect is assumed to be based on the influences of the limbic system on motor control^104,105^. The integration of the different central structures during adaptive motor processes leads to the conceivable and even likely assumption that also other internal and external inputs, which enter the control circuitries, might influence the adaptive motor control processes. The influence of health complaints on muscle function is reported for several indications, e.g., for infections as COVID-19^106^, post-infectious diseases^107^, CFS/ME^108,109^, cancer^110^, sarcopenia^111,112^, hormonal dysfunctions^113,114^ or fibromyalgia^115^. Thereby, possible nociception or other disturbing inputs might function as interferences in the complex motor control processes. We assume an impairment of the AFiso_max_ thereby.

When a muscle gets unstable under certain circumstances this could lead to a destabilization of joints especially when they are under strain. A higher vulnerability regarding joint complaints or even injuries might arise in that process. In contrast to measure maximal forces as usual, the assessment of the special parameter AFiso_max_ could provide a novel approach to understand injuries or orthopedic pathomechanisms.

Summarizing, the results highlight not only the suggested possibility of measuring a special adaptive neuromuscular control by the AF but might also deliver an approach for investigating the neuromuscular system regarding disturbances in the control circuits. The literature speaks for a complex control cascade as well as parallel working processes between the central areas characterized by oscillations which are involved in the control of the spatio-temporal structure of motor output. In an undisturbed healthy neuromuscular system those complex control processes should enable the participant to adapt adequately to the external force stimuli.

## Conclusion

The present study showed different adaptive motor outputs in reaction to neutral, pleasant and disgusting odours in healthy persons. Thereby, the findings might help reducing the lack of knowledge concerning the influence of olfaction on the motor control of respiratory-independent muscles. Assuming that the AF in reaction to neutral and pleasant odours reflect “normal” muscle function, the AF patterns during disgusting odours are interpreted as a disturbance of the neuromuscular control due to the unpleasant olfactory stimuli. Based on the presented preliminary results, it is suggested that the length tension control of muscles is affected thereby. Therefore, the isometric holding function including the mechanical muscle oscillations might be one or even the decisive parameter characterizing a well-functioning neuromuscular control of AF action. It is hypothesized that measuring the AF, in particular the parameters AFiso_max_ and AFosc, might be a suitable diagnostic tool to assess the functionality of neuromuscular control.

Based on the complex neuronal control, which is assumed to underlie the processing of AF, it is presumed that also other inputs as mental stress (negative emotions), nociception of joints or tissues or the like might influence the AF as shown here for disgusting odours. If this hypothesis could be verified by further investigations, this might offer the possibility to use the measurement of AF as an individual diagnostic tool. The MMT is already used since decades^39,49^. However, due to the reasonable criticism of subjectivity, some skepticism concerning the AF tested by the MMT remains in different fields for which it might have potential. The acceptance could be improved by objectification using appropriate devices. The assessment and recording of the manually tested AF are necessary to secure a reliable and valid force profile of the tester. Because of the preliminary character of the present study further measurements with an enlarged data base are needed. In a next step, the AF in reaction to emotions and nociception should verify further evidence of the assumed responses of the neuromuscular control to different inputs.

## Supplementary Information

Supplementary Tables.

## Data Availability

Data is contained within the article or supplementary material.

## References

[1] Ferdenzi C, Fournel A, Thévenet M, Coppin G, Bensafi M (2015). Viewing olfactory affective responses through the sniff prism: Effect of perceptual dimensions and age on olfactomotor responses to odors. Front. Psychol..

[2] Johnson BN, Mainland JD, Sobel N (2003). Rapid olfactory processing implicates subcortical control of an olfactomotor system. J. Neurophysiol..

[3] Doty RL (2001). Olfaction. Annu. Rev. Psychol..

[4] Pinto JM (2011). Olfaction. Proc. Am. Thorac. Soc..

[5] Gire DH (2013). Temporal processing in the olfactory system: Can we see a smell?. Neuron.

[6] Shepherd, G. M. *The Synaptic Organization of the Brain* (Oxford University Press, 2004).

[7] Morecraft, R. J. & Tanjii, J. Cingulofrontal interactions and the cingulate motor areas. In *Cingulate Neurobiology and Disease* (ed. Vogt, B.) 113–144 (Oxford University Press, 2009).

[8] Sagaspe P, Schwartz S, Vuilleumier P (2011). Fear and stop: A role for the amygdala in motor inhibition by emotional signals. Neuroimage.

[9] LeDoux JE (2000). Emotion circuits in the brain. Annu. Rev. Neurosci..

[10] Warren DW, Walker JC, Drake AF, Lutz RW (1994). Effects of odorants and irritants on respiratory behavior. Laryngoscope.

[11] Walker JC (2001). Human responses to propionic acid. II. Quantification of breathing responses and their relationship to perception. Chem. Senses.

[12] Frank RA, Dulay MF, Gesteland RC (2003). Assessment of the Sniff Magnitude Test as a clinical test of olfactory function. Physiol. Behav..

[13] Bensafi M (2003). Olfactomotor activity during imagery mimics that during perception. Nat. Neurosci..

[14] Sobel N (1998). Odorant-induced and sniff-induced activation in the cerebellum of the human. J. Neurosci..

[15] Sakuma K, Nakashima K, Takahashi K (1996). Olfactory evoked potentials in Parkinson’s disease, Alzheimer’s disease and anosmic patients. Psychiatry Clin. Neurosci..

[16] Derjean D (2010). A novel neural substrate for the transformation of olfactory inputs into motor output. PLoS Biol..

[17] Ehrlichman H, Brown S, Zhu J, Warrenburg S (1995). Startle reflex modulation during exposure to pleasant and unpleasant odors. Psychophysiology.

[18] Sakamoto Y (2012). Fall prevention using olfactory stimulation with lavender odor in elderly nursing home residents: A randomized controlled trial. J. Am. Geriatr. Soc..

[19] Smith CJ, Scott SM, Ryan BA (1999). Cardiovascular effects of odors. Toxicol. Ind. Health.

[20] Nagai M, Wada M, Usui N, Tanaka A, Hasebe Y (2000). Pleasant odors attenuate the blood pressure increase during rhythmic handgrip in humans. Neurosci. Lett..

[21] Sowndhararajan K, Kim S (2016). Influence of fragrances on human psychophysiological activity: With special reference to human electroencephalographic response. Sci. Pharm..

[22] Baron RA, Thomley J (1994). A Whiff of reality: Positive affect as a potential mediator of the effects of pleasant fragrances on task performance and helping. Environ. Behav..

[23] Glass ST, Lingg E, Heuberger E (2014). Do ambient urban odors evoke basic emotions?. Front. Psychol..

[24] Schiffman SS (1998). Livestock odors: Implications for human health and well-being. J. Anim. Sci..

[25] Schaefer LV, Bittmann FN (2021). Paired personal interaction reveals objective differences between pushing and holding isometric muscle action. PLoS ONE.

[26] Schaefer LV, Bittmann FN (2017). Are there two forms of isometric muscle action? Results of the experimental study support a distinction between a holding and a pushing isometric muscle function. BMC Sports Sci. Med. Rehabil..

[27] Hunter SK, Ryan DL, Ortega JD, Enoka RM (2002). Task differences with the same load torque alter the endurance time of submaximal fatiguing contractions in humans. J. Neurophysiol..

[28] Rudroff T, Justice JN, Holmes MR, Matthews SD, Enoka RM (2011). Muscle activity and time to task failure differ with load compliance and target force for elbow flexor muscles. J. Appl. Physiol..

[29] Rudroff T, Barry BK, Stone AL, Barry CJ, Enoka RM (2007). Accessory muscle activity contributes to the variation in time to task failure for different arm postures and loads. J. Appl. Physiol..

[30] Rudroff T (2013). PET/CT imaging of age- and task-associated differences in muscle activity during fatiguing contractions. J. Appl. Physiol..

[31] Garner JC, Blackburn T, Weimar W, Campbell B (2008). Comparison of electromyographic activity during eccentrically versus concentrically loaded isometric contractions. J. Electromyogr. Kinesiol..

[32] Enoka RM (1996). Eccentric contractions require unique activation strategies by the nervous system. J. Appl. Physiol..

[33] Duchateau J, Enoka RM (2008). Neural control of shortening and lengthening contractions: Influence of task constraints: Shortening and lengthening contractions. J. Physiol..

[34] Duchateau J, Enoka RM (2016). Neural control of lengthening contractions. J. Exp. Biol..

[35] Duchateau J, Baudry S (2014). Insights into the neural control of eccentric contractions. J. Appl. Physiol..

[36] Pflüger, H. J. & Sillar, K. Motor control. In *Neurosciences—From Molecule to Behavior: A University Textbook* (eds Galizia, C. G. & Lledo, P.-M.) 479–524 (Springer, 2013).

[37] Huggenberger S (2019). Neuroanatomie des Menschen: mit 202 größtenteils farbigen Abbildungen.

[38] Dech S, Bittmann FN, Schaefer LV (2021). Assessment of the adaptive force of elbow extensors in healthy subjects quantified by a novel pneumatically driven measurement system with considerations of its quality criteria. Diagnostics.

[39] Bittmann FN, Dech S, Aehle M, Schaefer LV (2020). Manual muscle testing—Force profiles and their reproducibility. Diagnostics.

[40] Hoff M, Schaefer L, Heinke N, Bittmann F (2015). Report on Adaptive Force, a specific neuromuscular function. Eur. J. Transl. Myol..

[41] Schaefer L, Hoff M, Bittmann F (2017). Measuring system and method of determining the Adaptive Force. Eur. J. Transl. Myol..

[42] Caligiore D (2017). Consensus paper: Towards a systems-level view of cerebellar function: The interplay between cerebellum, basal ganglia, and cortex. Cerebellum.

[43] Ishikawa T, Tomatsu S, Izawa J, Kakei S (2016). The cerebro-cerebellum: Could it be loci of forward models?. Neurosci. Res..

[44] Zobel S (2010). Involvement of the human ventrolateral thalamus in olfaction. J. Neurol..

[45] Soudry Y, Lemogne C, Malinvaud D, Consoli S-M, Bonfils P (2011). Olfactory system and emotion: Common substrates. Eur. Ann. Otorhinolaryngol. Head Neck Dis..

[46] Delplanque S (2008). Emotional processing of odors: Evidence for a nonlinear relation between pleasantness and familiarity evaluations. Chem. Senses.

[47] Weber ST, Heuberger E (2008). The impact of natural odors on affective states in humans. Chem. Senses.

[48] Vanderwolf CH (2001). The hippocampus as an olfacto-motor mechanism: Were the classical anatomists right after all?. Behav. Brain Res..

[49] Conable KM, Rosner AL (2011). A narrative review of manual muscle testing and implications for muscle testing research. J. Chiropractic Med..

[50] Schaefer LV, Torick AH, Matuschek H, Holschneider M, Bittmann FN (2014). Synchronization of muscular oscillations between two subjects during isometric interaction. Eur. J. Transl. Myol..

[51] Schaefer LV, Bittmann FN (2018). Coherent behavior of neuromuscular oscillations between isometrically interacting subjects: Experimental study utilizing wavelet coherence analysis of mechanomyographic and mechanotendographic signals. Sci. Rep..

[52] McAuley JH (2000). Physiological and pathological tremors and rhythmic central motor control. Brain.

[53] Beck, T. *Applications of Mechanomyography for Examining Muscle Function* (Transworld Research Network, 2010). https://issuu.com/researchsignpost/docs/beck/63.

[54] Rosenbaum DA (2010). Human Motor Control.

[55] Galizia, C. G. & Lledo, P.-M. *Neurosciences—From Molecule to Behavior: A University Textbook* (Springer, 2013). 10.1007/978-3-642-10769-6.

[56] Rothwell JC (1987). Control of Human Voluntary Movement.

[57] Albus JS (1971). A theory of cerebellar function. Math. Biosci..

[58] Ashe J, Bushara K, Merchant H, de Lafuente V (2014). The olivo-cerebellar system as a neural clock. Neurobiology of Interval Timing.

[59] Lawrenson C (2018). The mystery of the cerebellum: Clues from experimental and clinical observations. Cerebellum Ataxias.

[60] Shadmehr R, Smith MA, Krakauer JW (2010). Error correction, sensory prediction, and adaptation in motor control. Annu. Rev. Neurosci..

[61] Ivry RB (2000). Exploring the role of the cerebellum in sensory anticipation and timing: Commentary on Tesche and Karhu. Hum. Brain Mapp..

[62] Bengtsson F, Ekerot C-F, Jörntell H (2011). In vivo analysis of inhibitory synaptic inputs and rebounds in deep cerebellar nuclear neurons. PLoS One.

[63] Lang EJ (2017). The roles of the olivocerebellar pathway in motor learning and motor control. A consensus paper. Cerebellum.

[64] Groenewegen HJ (2003). The basal ganglia and motor control. Neural Plast..

[65] Jörntell H (2017). Cerebellar physiology: Links between microcircuitry properties and sensorimotor functions: Cerebellar physiology. J. Physiol..

[66] Vogt BA, Finch DM, Olson CR (1992). Functional heterogeneity in cingulate cortex: The anterior executive and posterior evaluative regions. Cereb. Cortex.

[67] Doya K (2000). Complementary roles of basal ganglia and cerebellum in learning and motor control. Curr. Opin. Neurobiol..

[68] Wu T, Hallett M (2013). The cerebellum in Parkinson’s disease. Brain.

[69] Pelzer EA (2013). Cerebellar networks with basal ganglia: Feasibility for tracking cerebello-pallidal and subthalamo-cerebellar projections in the human brain. Eur. J. Neurosci..

[70] Gerfen, C. R. & Wilson, C. J. Chapter II The basal ganglia. In *Handbook of Chemical Neuroanatomy* vol. 12 (eds Swanson, L. W., Björklund, A. & Hökfelt, A.) 371–468 (Elsevier, 1996). 10.1016/S0924-8196(96)80004-2.

[71] Wise SP, Murray EA, Gerfen CR (1996). The frontal cortex-basal ganglia system in primates. Crit. Rev. Neurobiol..

[72] O’Halloran CJ, Kinsella GJ, Storey E (2012). The cerebellum and neuropsychological functioning: A critical review. J. Clin. Exp. Neuropsychol..

[73] Schlerf JE, Galea JM, Bastian AJ, Celnik PA (2012). Dynamic modulation of cerebellar excitability for abrupt, but not gradual, visuomotor adaptation. J. Neurosci..

[74] Jueptner M (1997). The relevance of sensory input for the cerebellar control of movements. Neuroimage.

[75] Welsh JP, Lang EJ, Suglhara I, Llinás R (1995). Dynamic organization of motor control within the olivocerebellar system. Nature.

[76] Vitek JL, Ashe J, DeLong MR, Alexander GE (1994). Physiologic properties and somatotopic organization of the primate motor thalamus. J. Neurophysiol..

[77] Sherman S (2006). Thalamus. Scholarpedia.

[78] Haber SN, Calzavara R (2009). The cortico-basal ganglia integrative network: The role of the thalamus. Brain Res. Bull..

[79] Sommer MA (2003). The role of the thalamus in motor control. Curr. Opin. Neurobiol..

[80] Vogt BA, Nimchinsky EA, Vogt LJ, Hof PR (1995). Human cingulate cortex: Surface features, flat maps, and cytoarchitecture. J. Comp. Neurol..

[81] Pruszynski JA, Scott SH (2012). Optimal feedback control and the long-latency stretch response. Exp. Brain Res..

[82] Dickenson AH (2002). Editorial I. Br. J. Anaesth..

[83] Bueti D, Walsh V, Frith C, Rees G (2008). Different brain circuits underlie motor and perceptual representations of temporal intervals. J. Cogn. Neurosci..

[84] D’Angelo, E. Chapter 6 - Physiology of the cerebellum. In *Handbook of Clinical Neurology* vol. 154 (eds Manto, M. & Huisman, T. A. G. M.) 85–108 (Elsevier, 2018). 10.1016/B978-0-444-63956-1.00006-0. 10.1016/B978-0-444-63956-1.00006-0 29903454

[85] Rao SM (1997). Distributed neural systems underlying the timing of movements. J. Neurosci..

[86] Lang EJ, Sugihara I, Welsh JP, Llinás R (1999). Patterns of spontaneous Purkinje cell complex spike activity in the awake rat. J. Neurosci..

[87] Manning CD, Tolhurst SA, Bawa P (2012). Proprioceptive reaction times and long-latency reflexes in humans. Exp. Brain Res..

[88] Gross J (2002). The neural basis of intermittent motor control in humans. Proc. Natl. Acad. Sci..

[89] Bays PM, Wolpert DM (2007). Computational principles of sensorimotor control that minimize uncertainty and variability: Computational principles of sensorimotor control. J. Physiol..

[90] Johansson, R. S. How is grasping modified by somatosensory input? In *Motor Control: Concepts and Issues* (ed. Humphrey, D. R. & Freund, H.-J.) 331–355 (Wiley, 1991).

[91] Todorov E (2004). Optimality principles in sensorimotor control. Nat. Neurosci..

[92] Ivry RB (1996). The representation of temporal information in perception and motor control. Curr. Opin. Neurobiol..

[93] Lewis PA, Miall RC (2003). Brain activation patterns during measurement of sub- and supra-second intervals. Neuropsychologia.

[94] Farmer SF, Bremner FD, Halliday DM, Rosenberg JR, Stephens JA (1993). The frequency content of common synaptic inputs to motoneurones studied during voluntary isometric contraction in man. J. Physiol..

[95] Kakuda N, Nagaoka M, Wessberg J (1999). Common modulation of motor unit pairs during slow wrist movement in man. J. Physiol..

[96] Semmler JG, Kornatz KW, Dinenno DV, Zhou S, Enoka RM (2002). Motor unit synchronisation is enhanced during slow lengthening contractions of a hand muscle. J. Physiol..

[97] Tass P (1998). Detection of n:m phase locking from noisy data: Application to magnetoencephalography. Phys. Rev. Lett..

[98] Raethjen J (2002). Corticomuscular coherence in the 6–15 Hz band: Is the cortex involved in the generation of physiologic tremor?. Exp. Brain Res..

[99] Burkhardt M (2006). Aktivitäten des sensor-motorischen Kortex bei Patienten mit komplexem regionalen Schmerzsyndrom (CRPS).

[100] Paus T, Sipila PK, Strafella AP (2001). Synchronization of neuronal activity in the human primary motor cortex by transcranial magnetic stimulation: An EEG study. J. Neurophysiol..

[101] Başar E (2012). A review of alpha activity in integrative brain function: Fundamental physiology, sensory coding, cognition and pathology. Int. J. Psychophysiol..

[102] Engen T (1982). The Perception of Odors.

[103] Gibbons B (1987). The intimate sense of smell. Natl. Geogr..

[104] Holstege, G. Chapter 14 Descending motor pathways and the spinal motor system: Limbic and non-limbic components. In *Progress in Brain Research* vol. 87 (ed. Holstege, G.) 307–421 (Elsevier, 1991). 10.1016/S0079-6123(08)63057-5. 10.1016/s0079-6123(08)63057-5 1678191

[105] Mogenson G, Jones D, Yim C (1980). From motivation to action: Functional interface between the limbic system and the motor system. Prog. Neurobiol..

[106] Angelini C, Siciliano G (2020). Neuromuscular diseases and Covid-19: Advices from scientific societies and early observations in Italy. Eur. J. Transl. Myol..

[107] Hickie I (2006). Post-infective and chronic fatigue syndromes precipitated by viral and non-viral pathogens: Prospective cohort study. BMJ.

[108] Nacul LC, Mudie K, Kingdon CC, Clark TG, Lacerda EM (2018). Hand grip strength as a clinical biomarker for ME/CFS and disease severity. Front. Neurol..

[109] Pietrangelo T, Fulle S, Coscia F, Gigliotti PV, Fanò-Illic G (2018). Old muscle in young body: An aphorism describing the Chronic Fatigue Syndrome. Eur. J. Transl. Myol..

[110] Dalise S, Tropea P, Galli L, Sbrana A, Chisari C (2020). Muscle function impairment in cancer patients in pre-cachexia stage. Eur. J. Transl. Myol..

[111] Edmunds KJ (2016). Quantitative computed tomography and image analysis for advanced muscle assessment. Eur. J. Transl. Myol..

[112] Šarabon N, Smajla D, Kozinc Ž, Kern H (2020). Speed-power based training in the elderly and its potential for daily movement function enhancement. Eur. J. Transl. Myol..

[113] Duyff RF (2000). Neuromuscular findings in thyroid dysfunction: A prospective clinical and electrodiagnostic study. J. Neurol. Neurosurg. Psychiatry.

[114] Hajjar K, Hagenacker T (2017). Neuromuscular disorder as initial manifestation of secondary hyperparathyroidism—A case report. Eur. J. Transl. Myol..

[115] Watson NF, Buchwald D, Goldberg J, Noonan C, Ellenbogen RG (2009). Neurologic signs and symptoms in fibromyalgia. Arthritis Rheumatol..

