# Supplementary material for: Disgusting odours affect the characteristics of the Adaptive Force in contrast to neutral and pleasant odours
Source: Sci Rep. 2021 Aug 12;11:16410. doi: 10.1038/s41598-021-95759-0 (PMC8361115; doi:10.1038/s41598-021-95759-0)
Supplement: Supplementary file 1 — Supplementary Tables. [file 41598_2021_95759_MOESM1_ESM.pdf]

Supplementary material

Article

Motor control and olfaction – Influence of pleasant and disgusting odors on the Adaptive Force

Schaefer LV, Dech S, Aehle M, Bittmann FN

Content

Table S1: Anthropometric data ... 2

Table S2. Information of tester, tested muscle and MMT assessment ... 2

Table S3: Maximum Adaptive Force (AF_{max}) ... 3

Table S4: Maximum isometric Adaptive Force ($AFiso_{max}$) ... 4

Table S5: Adaptive Force at onset of oscillations (AFosc) ... 5

Table S6: Slope ... 6

Table S1: Anthropometric data

Table S1. Anthropometric data of each participant: gender (male = 0; female = 1), age (years), height (cm), mass (kg)).

participant	gender	age (yrs.)	height (cm)	mass (kg)
1	0	31	189	83
2	1	26	152	70
3	1	26	152	70
4	1	24	159	55
5	1	27	167	54
6	1	57	162	70
7	0	44	184	84
8	0	27	184	68
9	1	46	173	77
10	0	25	187	81

Table S2. Information of tester, tested muscle and MMT assessment

Table S2. Information of tester (1 = female tester, 2 = male tester), tested muscle (1 = rectus femoris muscle; 2 = biceps brachii muscle) and assessment of MMT by the tester ("stable" = 1 ; "unstable" = 0) for each participant. (neut = neutral; pleas = pleasant; disg = disgusting; _1 to _3 refers to number of trial)

			Assessment of MMT								
Participant	tester	tested muscle	neut_1	neut_2	neut_3	pleas_1	pleas_2	pleas_3	disg_1	disg_2	disg_3
1	2	1	1	1	1	1	1	1	0	0	0
2	2	2	1	1	1	1	1	-	0	0	0
3	2	1	1	1	1	1	1	-	0	0	-
4	1	2	0	1	1	1	1	1	0	0	0
5	1	1	1	1	1	1	1	1	1	-*	0
6	2	1	1	1	1	1	1	0	0	0	0
7	1	1	1	1	-	1	1	-	0	0	-
8	1	1	1	1	-	1	1	1	0	0	-
9	1	1	1	1	-	1	1	1	0	0	-
10	1	1	1	-	-	1	1	-	0	0	-

* excluded because of technical problems.

Table S3: Maximum Adaptive Force (AF_{max})

Table S3. Maximum of Adaptive Force (AFmax (N)) of each trial (M1-M3), participant (n = 10) and odors (neutral, pleasant, disgusting). The arithmetic means (M), standard deviations (SD) and coefficients of variation (CV) are given per participant and for the group.

	**neutral**			**pleasant**			**disgusting**			**neutral**			**pleasant**			**disgusting**		
	**M1**	**M2**	**M3**	**M1**	**M2**	**M3**	**M1**	**M2**	**M3**	**M**	**SD**	**CV**	**M**	**SD**	**CV**	**M**	**SD**	**CV**
1	241.42	254.18	261.44	260.55	275.96	278.11	265.95	252.71	258.49	252.34	10.13	0.04	271.54	9.58	0.04	259.05	6.64	0.03
2	224.06	216.41	215.33	222.29	224.94	-	226.32	205.91	224.26	218.60	4.76	0.02	223.62	1.87	0.01	218.83	11.23	0.05
3	208.85	217.39	209.44	214.25	219.16	-	229.26	268.21	-	211.90	4.77	0.02	216.70	3.47	0.02	248.73	27.54	0.11
4	241.52	165.42	160.87	159.42	188.60	178.53	230.47	176.24	150.00	189.27	45.31	0.24	175.52	14.82	0.08	185.57	41.04	0.22
5	174.08	143.69	174.90	180.65	158.13	151.17	172.41	-	200.54	164.22	17.79	0.11	163.32	15.41	0.09	186.48	19.89	0.11
6	207.90	201.24	203.36	207.43	207.53	213.66	163.34	191.91	190.27	204.17	3.40	0.02	209.54	3.57	0.02	181.84	16.04	0.09
7	232.08	227.50	-	230.68	233.53	-	246.77	251.10	-	229.79	3.24	0.01	232.10	2.01	0.01	248.93	3.06	0.01
8	164.71	139.69	-	170.30	136.75	198.36	246.82	237.11	-	152.20	17.69	0.12	168.47	30.84	0.18	241.96	6.87	0.03
9	113.40	146.46	-	132.83	137.32	182.86	183.84	158.14	-	129.93	23.38	0.18	151.00	27.68	0.18	170.99	18.17	0.11
10	143.22	-	-	194.92	215.53	-	241.23	271.05	-	143.22	-	-	205.23	14.57	0.07	256.14	21.09	0.08
									M	189.56			201.70			219.85		
									SD	40.63			37.14			35.19		
									CV	0.21			0.18			0.16		

Table S4: Maximum isometric Adaptive Force ($AFiso_{max}$)

Table S4. Maximum isometric Adaptive Force ($AFiso_{max}$ (N)) of each trial (M1-M3), participant (n = 10) and odors (neutral, pleasant, disgusting). The arithmetic means (M), standard deviations (SD) and coefficients of variation (CV) are given per participant and for the group.

	neutral			pleasant			disgusting			neutral			pleasant			disgusting		
	**M1**	**M2**	**M3**	**M1**	**M2**	**M3**	**M1**	**M2**	**M3**	**M**	**SD**	**CV**	**M**	**SD**	**CV**	**M**	**SD**	**CV**
1	241.42	254.18	261.44	260.55	275.96	252.41	173.83	181.68	186.49	252.35	10.13	0.04	262.97	11.96	0.05	180.67	6.39	0.04
2	222.59	216.41	215.33	222.29	211.01	-	180.60	124.88	82.60	218.11	3.92	0.02	216.65	7.98	0.04	129.36	49.15	0.38
3	208.85	217.39	209.44	214.25	219.16	-	77.30	120.96	-	211.90	4.77	0.02	216.70	3.47	0.02	99.13	30.87	0.31
4	175.51	165.42	160.87	159.42	188.60	178.53	166.07	110.95	75.50	167.27	7.49	0.04	175.52	14.82	0.08	117.51	45.64	0.39
5	174.08	143.69	174.90	173.37	151.22	151.17	172.41	-	100.79	164.22	17.79	0.11	158.59	12.80	0.08	136.60	50.64	0.37
6	207.90	160.16	203.36	207.43	207.53	171.04	86.50	103.60	70.07	190.47	26.35	0.14	195.33	21.04	0.11	86.72	16.77	0.19
7	232.08	227.50	-	230.68	214.76	-	135.70	162.77	-	229.79	3.24	0.01	222.72	11.26	0.05	149.23	19.14	0.13
8	164.71	139.69	-	170.30	136.75	198.36	162.55	160.69	-	152.20	17.69	0.12	168.47	30.84	0.18	161.62	1.32	0.01
9	113.40	146.46	-	132.83	137.32	182.86	109.97	95.75	-	129.93	23.38	0.18	151.00	27.68	0.18	102.86	10.06	0.10
10	143.22	-	-	194.92	215.53	-	135.87	165.89	-	143.22	-	-	205.23	14.57	0.07	150.88	21.23	0.14
									M	185.95			197.32			131.46		
									SD	40.80			34.52			30.03		
									CV	0.22			0.17			0.23		

Table S5: Adaptive Force at onset of oscillations (AFosc)

Table S5. Adaptive Force at the onset of oscillations (AFosc (N)) of each trial (M1-M3), participant (n = 10) and odors (neutral, pleasant, disgusting). The arithmetic means (M), standard deviations (SD) and coefficients of variation (CV) are given per participant and for the group.

	**neutral**			**pleasant**			**disgusting**			**neutral**			**pleasant**			**disgusting**		
	**M1**	**M2**	**M3**	**M1**	**M2**	**M3**	**M1**	**M2**	**M3**	**M**	**SD**	**CV**	**M**	**SD**	**CV**	**M**	**SD**	**CV**
1	199.54	210.52	222.59	177.76	233.77	154.80	265.95	252.71	249.96	210.88	11.53	0.05	188.78	40.62	0.22	256.20	8.55	0.03
2	221.61	190.41	181.78	163.43	211.60	-	226.32	205.91	213.27	197.93	20.95	0.11	187.52	34.06	0.18	215.17	10.33	0.05
3	164.77	186.25	186.78	160.49	185.41	-	229.26	259.87	-	179.27	12.56	0.07	172.95	17.62	0.10	244.56	21.64	0.09
4	177.10	118.20	90.05	119.60	170.97	154.07	229.90	140.24	145.98	128.45	44.42	0.35	148.21	26.18	0.18	172.04	50.19	0.29
5	126.40	96.26	146.75	154.73	133.68	121.56	165.21	-	154.13	123.14	25.40	0.21	136.66	16.78	0.12	159.67	7.83	0.05
6	152.97	160.55	176.23	177.84	178.65	196.18	159.72	189.63	168.29	163.25	11.86	0.07	184.22	10.36	0.06	172.55	15.40	0.09
7	166.00	193.24	-	180.93	186.60	-	246.77	249.64	-	179.62	19.26	0.11	183.76	4.01	0.02	248.21	2.03	0.01
8	118.31	100.85	-	135.57	88.49	125.76	244.56	225.92	-	109.58	12.35	0.11	116.61	24.84	0.21	235.24	13.18	0.06
9	82.11	84.76	-	106.14	95.06	130.67	174.62	151.76	-	83.43	1.87	0.02	110.62	18.22	0.16	163.19	16.16	0.10
10	81.42	-	-	144.89	98.30	-	141.95	257.71	-	81.42	-	-	121.59	32.95	0.27	199.83	81.85	0.41
									M	145.70			155.09			206.67		
									SD	46.76			31.87			38.03		
									CV	0.32			0.21			0.18		

Table S6: Slope

Table S6. Slope lg(N/s) of each trial (M1-M3), participant (n = 10) and odors (neutral, pleasant, disgusting). The arithmetic means (M), standard deviations (SD) and coefficients of variation (CV) are given per participant and for the group.

	**neutral**			**pleasant**			**disgusting**			**neutral**			**pleasant**			**disgusting**		
	**M1**	**M2**	**M3**	**M1**	**M2**	**M3**	**M1**	**M2**	**M3**	**M**	**SD**	**CV**	**M**	**SD**	**CV**	**M**	**SD**	**CV**
1	1.930	2.140	2.110	1.970	2.070	2.020	2.080	2.160	2.150	2.06	0.11	0.06	2.02	0.05	0.02	2.13	0.04	0.02
2	2.061	1.840	2.086	2.012	2.076	-	2.090	1.950	2.013	2.00	0.14	0.07	2.04	0.04	0.02	2.02	0.07	0.03
3	1.997	1.953	1.876	2.104	2.207	-	1.915	1.980	-	1.94	0.06	0.03	2.16	0.07	0.03	1.95	0.05	0.02
4	2.075	1.904	-	1.978	2.050	1.985	2.040	2.026	1.768	1.99	0.12	0.06	2.00	0.04	0.02	1.94	0.15	0.08
5	1.879	1.805	1.928	1.982	-	-	1.914	-	1.918	1.87	0.06	0.03	1.98	-	-	1.92	0.00	0.00
6	1.956	2.152	2.051	1.823	2.038	2.116	1.487	1.916	1.866	2.05	0.10	0.05	1.99	0.15	0.08	1.76	0.23	0.13
7	2.155	2.165	-	1.977	2.101	-	2.097	2.204	-	2.16	0.01	0.00	2.04	0.09	0.04	2.15	0.08	0.04
8	-	-	-	-	-	1.965	1.882	1.998	-	-	-	-	1.97	-	-	1.94	0.08	0.04
9	1.689	1.762	-	1.887	1.857	1.919	1.862	1.856	-	1.73	0.05	0.03	1.89	0.03	0.02	1.86	0.00	0.00
10	-	-	-	1.886	1.751	-	1.918	1.952	-	-	-	-	1.82	0.10	0.05	1.94	0.02	0.01
									M	1.975			1.991			1.960		
									SD	0.132			0.091			0.117		
									CV	0.067			0.046			0.060		
